# Diagnostic Performance of Radiofrequency Echographic Multi Spectrometry Compared with Dual-Energy X-Ray Absorptiometry: A Real-World Study

**DOI:** 10.3390/diagnostics16213453

**Published:** 2026-10-22

**Authors:** Isabella Nardone, Sium Wolde Sellasie, Simona Zaccaria, Marina Passeri, Stefania Falcone, Chiara Pecchioli, Laura Giurato, Luigi Uccioli

**Affiliations:** 1 Division of Endocrinology and Diabetes, CTO Andrea Alesini Hospital, Department of Surgical Sciences, University of Rome Tor Vergata, 00133 Rome, Italy; 2 PhD School of Applied Medical-Surgical Sciences, University of Rome Tor Vergata, 00133 Rome, Italy

**Keywords:** REMS, DXA, osteoporosis, bone fragility

## Abstract

**Background:** Radiofrequency Echographic Multi-Spectrometry (REMS) is an ultrasound-based technique for the assessment of bone mineral density (BMD) that has been proposed as an alternative to dual-energy X ray absorptiometry (DXA) for the diagnosis of osteoporosis. Although previous studies have reported high diagnostic concordance between REMS and DXA, evidence from real-world settings remains limited. The aim of this study was to evaluate the diagnostic performance and agreement between REMS and DXA in a clinical population undergoing BMD assessment. **Methods:** In this retrospective observational study, 145 subjects undergoing BMD evaluation were assessed with both REMS and DXA. Complete paired examinations were available for 142 patients at the lumbar spine and 134 at the femoral neck. DXA was used as the reference standard. Diagnostic performance of REMS was evaluated by calculating sensitivity, specificity, positive predictive value (PPV), and negative predictive value (NPV). Agreement between REMS and DXA diagnostic classifications was assessed using linearly weighted Cohen’s kappa. Mean REMS and DXA measurements were compared using paired Student’s t-tests, whereas agreement between continuous BMD measurements was assessed using Bland–Altman analysis. Receiver operating characteristic (ROC) analysis was performed to evaluate the discriminative performance of REMS T-scores. **Results:** At the lumbar spine, REMS showed a sensitivity of 50.0% (95% CI: 37.5–62.5), specificity of 65.5% (95% CI: 54.8–74.8), PPV of 50.0% (95% CI: 37.5–62.5), and NPV of 65.5% (95% CI: 54.8–74.8). At the femoral neck, sensitivity was 40.0% (95% CI: 28.1–53.2), specificity 70.9% (95% CI: 60.1–79.7), PPV 48.9% (95% CI: 35.0–63.0), and NPV 62.9% (95% CI: 52.5–72.2). ROC analysis showed limited discriminatory performance for DXA-defined osteoporosis, with AUC values of 0.610 at the lumbar spine and 0.623 at the femoral neck. Linearly weighted Cohen’s kappa values were 0.225 and 0.183, respectively, indicating limited agreement in diagnostic classification. No statistically significant differences in mean BMD or T-score values were detected between the two techniques. **Conclusions**: In this real-world retrospective cohort, REMS and DXA showed no statistically significant differences in mean BMD or T-score values, but agreement in individual diagnostic classification was limited. These results suggest that REMS may have a complementary role alongside DXA rather than serving as a standalone screening method for DXA-defined osteoporosis in this cohort. Further prospective studies are needed to clarify the clinical significance of REMS–DXA discordant cases and their relationship with fracture risk.

## 1. Introduction

Osteoporosis is a major public health issue characterized by reduced bone mass and deterioration of bone microarchitecture, leading to increased skeletal fragility and higher risk of fractures. Fragility fractures represent a major cause of morbidity, disability, reduced quality of life, and healthcare costs worldwide [1,2,3,4]. Early identification of individuals at increased fracture risk is therefore essential to implement appropriate preventive and therapeutic strategies. Dual-energy X-ray absorptiometry (DXA) is currently considered the reference standard for the assessment of bone mineral density (BMD). According to the World Health Organization criteria, osteoporosis is defined by a T-score ≤ −2.5 standard deviations (SDs), measured by DXA at the lumbar spine, femoral neck, or total hip. DXA is widely used in clinical practice because of its good precision, relatively low radiation exposure, and well-established predictive value for fracture risk [5,6,7]. However, DXA has several limitations in daily clinical practice. First, access to DXA may be limited in some healthcare settings, often resulting in long waiting lists and making timely follow-up challenging. Second, DXA measurements may be affected by structural changes such as spinal osteoarthritis, osteophytes, vertebral deformities, or vascular calcifications, which may lead to an overestimation of BMD, particularly at the lumbar spine [8,9]. Third, although radiation exposure is low, DXA still involves the use of ionizing radiation. These concerns have stimulated growing interest in alternative or complementary techniques for the assessment of bone health. Radiofrequency Echographic Multi-Spectrometry (REMS) [10,11,12,13,14,15] is a relatively new ultrasound (US)-based technique that estimates BMD by analyzing unfiltered radiofrequency US signals acquired during echographic scans of skeletal sites. One of the main advantages of REMS is that it does not involve ionizing radiation and can be performed using portable devices, potentially expanding access to bone health assessment across different clinical settings [16]. Previous validation studies have evaluated the performance of REMS in comparison with DXA. Many of these studies have reported high levels of diagnostic agreement between the two techniques, with concordance rates often exceeding 90% for the classification of normal bone density, osteopenia, and osteoporosis. These findings suggest that REMS may represent a promising tool for osteoporosis screening and diagnosis. However, most available evidence derives from controlled validation studies conducted in highly selected populations and under standardized acquisition conditions [17,18,19,20]. In these settings, patients with conditions that may interfere with BMD measurements are often excluded, and examinations are frequently performed by highly experienced operators. As a result, the diagnostic performance of REMS observed in validation studies may not fully reflect its performance and applicability in routine clinical practice. On the other hand, evidence on the performance of REMS in real-world clinical settings and specific disease populations remains limited [21,22,23,24,25], and its reliability in everyday osteoporosis diagnosis and management is not yet fully established. Moreover, direct head-to-head comparisons between REMS and DXA in real-world clinical cohorts are still scarce [26,27,28,29,30]. In real-world practice, several factors may influence BMD measurements, including patient-related characteristics, comorbidities, degenerative changes, and differences in acquisition technique. For these reasons, evaluating the agreement between REMS and DXA in routine clinical settings is essential to better understand the practical applicability of REMS and the factors that may influence its diagnostic performance. To address this gap, the present study aims to assess the diagnostic concordance between REMS and DXA in a real-world population of patients referred to an endocrinology outpatient clinic for BMD evaluation.

## 2. Materials and Methods

### 2.1. Study Conduction

The present study was conducted according to the STROBE (STrengthening the Reporting of OBservational studies in Epidemiology) statement [31].

#### Institutional Setting and Case Selection

This retrospective observational study included subjects who underwent BMD assessment at the Endocrinology Unit of CTO Hospital in Rome between January 2024 and June 2025. The Endocrinology Unit of CTO “A. Alesini” Hospital (Rome, Italy) is a multidisciplinary clinic dedicated to the diagnosis and treatment of several endocrine diseases. Assessment of bone health is routinely prescribed for both clinical evaluation and screening for endocrine disease related complications. The medical records of all patients who underwent REMS at our center during the study period were retrospectively reviewed. Eligible patients were men aged ≥50 years and postmenopausal women aged ≥50 years who underwent both REMS and DXA on the same day at our center. Patients whose DXA examinations had been performed elsewhere or whose DXA records were unavailable were excluded. DXA was performed in accordance with international guideline recommendations [32]. Demographic and anthropometric data, including sex, age and BMI, were extracted from the clinical records. The study was conducted in accordance with the principles of the Declaration of Helsinki. Informed consent was obtained from patients for data collection. Data were analyzed in anonymized form.

### 2.2. BMD Assessment

BMD was measured using DXA (Hologic Discovery A) at the lumbar spine (L1–L4) and femoral neck. T-scores were calculated using the manufacturer’s reference database. BMD assessment using REMS was performed at the lumbar spine and femoral neck using a portable EchoS device (EchoStudio, Echolight, Lecce, Italy). REMS estimates BMD through spectral analysis of raw radiofrequency US signals obtained during echographic scans. The acquired spectral profiles are compared with reference models to estimate BMD and T-scores [13,17].

REMS and DXA examinations were performed on the same day. All examinations were performed by two trained operators, one dedicated to DXA and the other to REMS. Operator-specific intra- or inter-observer reproducibility was not assessed in this retrospective study. The study protocol did not include formal blinding between the index test (REMS) and the reference standard (DXA); therefore, the operators could not be considered formally blinded to the results of the other technique. Examinations affected by acquisition errors or unevaluable measurements were excluded from the corresponding site-specific analyses. The number of evaluable examinations was recorded separately for each technique and anatomical site. Comparisons between REMS and DXA were restricted to patients with evaluable measurements obtained using both techniques at the same anatomical site. Bone status was categorized according to T-score values into three diagnostic classes: normal bone density (T-score ≥ −1.0 SD), osteopenia (−2.5 < T-score < −1.0 SD), and osteoporosis (T-score ≤ −2.5 SD).

### 2.3. Statistical Analysis

Statistical analyses were performed using Jamovi (version 2.7.24) [33]. Continuous variables were expressed as mean ± SD, when distribution was parametric, whereas categorical variables were reported as absolute numbers and percentages. Mean BMD and T-score values obtained by DXA and REMS were compared using the paired Student’s *t*-test. Differences in the overall distribution of the three diagnostic categories (normal bone density, osteopenia, and osteoporosis) between REMS and DXA were evaluated using the Stuart–Maxwell test for marginal homogeneity. The diagnostic performance of REMS for identifying osteoporosis was evaluated using DXA as the reference standard for densitometric classification. For each anatomical site, 2 × 2 contingency tables were constructed using patients with evaluable paired REMS and DXA examinations. Osteoporosis was considered a positive result, whereas normal bone density and osteopenia were combined into a single negative category. Sensitivity, specificity, positive predictive value (PPV), and negative predictive value (NPV) were calculated from the corresponding true-positive, false-positive, true-negative, and false-negative counts. The denominator used for each analysis was the number of patients with complete data for the variables included in that analysis. Ninety-five percent confidence intervals for sensitivity, specificity, PPV, and NPV were calculated using the Wilson score method.

Agreement between REMS and DXA diagnostic classifications (normal bone density, osteopenia, and osteoporosis) was assessed using linearly weighted Cohen’s kappa statistics. Three-category (3 × 3) cross-tabulations of REMS and DXA classifications were also constructed for each anatomical site. Agreement between REMS and DXA BMD measurements as continuous variables was evaluated using Bland–Altman analysis. The mean difference (bias) was calculated as REMS minus DXA, and the 95% limits of agreement were calculated as the mean difference ± 1.96 standard deviations of the paired differences. Receiver operating characteristic (ROC) curve analysis was performed as an exploratory analysis to evaluate the discriminative performance of REMS T-scores for identifying DXA-defined osteoporosis. The area under the ROC curve (AUC) with 95% confidence intervals was calculated. The optimal REMS T-score cut-off for identifying DXA-defined osteoporosis was determined by the Youden index. The analysis was not intended to redefine the established diagnostic threshold for osteoporosis. A *p*-value < 0.05 was considered statistically significant.

## 3. Results

Medical records of 206 patients who underwent REMS examination at our center were reviewed, 19 were excluded because younger than 50 years, including 13 premenopausal women. A further 42 patients were excluded because DXA had been performed outside our center or DXA report was unavailable. The final study population comprised 145 patients (Supplementary Figure S1).

The mean age was 63.8 ± 8.3 years, 130 patients were female (89.7%), and the mean BMI was 24.5 ± 4.1 kg/m^2^. Previously acknowledged conditions potentially associated with reduced BMD were present in 34.4% of patients, including primary hyperparathyroidism (4.8%), Turner syndrome (3.4%), Klinefelter syndrome (2.8%), and cancer treatment-induced bone loss (23.4%) (Table 1).

Of the 145 patients included in the study, 143 lumbar spine REMS examinations and 144 lumbar spine DXA examinations were evaluable; 2 REMS and 1 DXA examinations were excluded. At the femoral neck, 139 REMS examinations and 138 DXA examinations were evaluable, with 6 REMS and 7 DXA examinations excluded. Consequently, 142 patients had complete paired REMS and DXA data at the lumbar spine and 134 at the femoral neck. Diagnostic category distributions were calculated using the number of evaluable examinations for each technique and anatomical site, whereas direct comparisons between REMS and DXA were performed using paired data.

Densitometric characteristics are summarized in Table 2. Mean lumbar spine BMD was 0.839 ± 0.146 g/cm^2^ by DXA and 0.816 ± 0.085 g/cm^2^ by REMS (*p* = 0.059). Mean lumbar T-score was −1.95 ± 1.31 by DXA and −2.15 ± 0.75 by REMS (*p* = 0.075). At the femoral neck, mean BMD was 0.618 ± 0.112 g/cm^2^ by DXA and 0.623 ± 0.087 g/cm^2^ by REMS (*p* = 0.653). Mean T-score was −2.12 ± 0.93 by DXA and −2.07 ± 0.77 by REMS (*p* = 0.541). No statistically significant differences were observed between mean DXA and REMS measurements for either BMD or T-score at the lumbar spine or femoral neck. According to DXA, osteoporosis was present in 59 subjects (41.0%) at the lumbar spine and in 56 subjects (40.6%) at the femoral neck. Osteopenia was identified in 63 subjects (43.8%) at the lumbar spine and in 68 subjects (49.3%) at the femoral neck. According to REMS, osteoporosis was diagnosed in 59 subjects (41.3%) at the lumbar spine and in 47 subjects (33.8%) at the femoral neck, whereas osteopenia was identified in 75 subjects (52.4%) and 76 subjects (54.7%), respectively. When the overall distribution across the three diagnostic categories was compared using paired evaluable examinations, a significant difference between REMS and DXA was observed at the lumbar spine (*p* = 0.005), whereas no significant difference was observed at the femoral neck (*p* = 0.408).

Diagnostic performance was evaluated in patients with complete paired REMS and DXA examinations (*n* = 142 at the lumbar spine and *n* = 134 at the femoral neck). The corresponding 2 × 2 contingency tables are provided in Supplementary Table S1. Using DXA as the reference for densitometric classification, false-positive classifications denote osteoporosis identified by REMS but not by DXA, whereas false-negative classifications denote osteoporosis identified by DXA but not by REMS. At the lumbar spine, 29 patients were correctly classified as osteoporotic by REMS, whereas 29 were classified as false positives and 29 as false negatives, with 55 true-negative classifications. These counts corresponded to a sensitivity of 50.0%, specificity of 65.5%, PPV of 50.0%, and NPV of 65.5%. At the femoral neck, 22 true-positive, 23 false-positive, 56 true-negative, and 33 false-negative classifications were observed, corresponding to a sensitivity of 40.0%, specificity of 70.9%, PPV of 48.9%, and NPV of 62.9% (Table 3).

**Figure 1:**
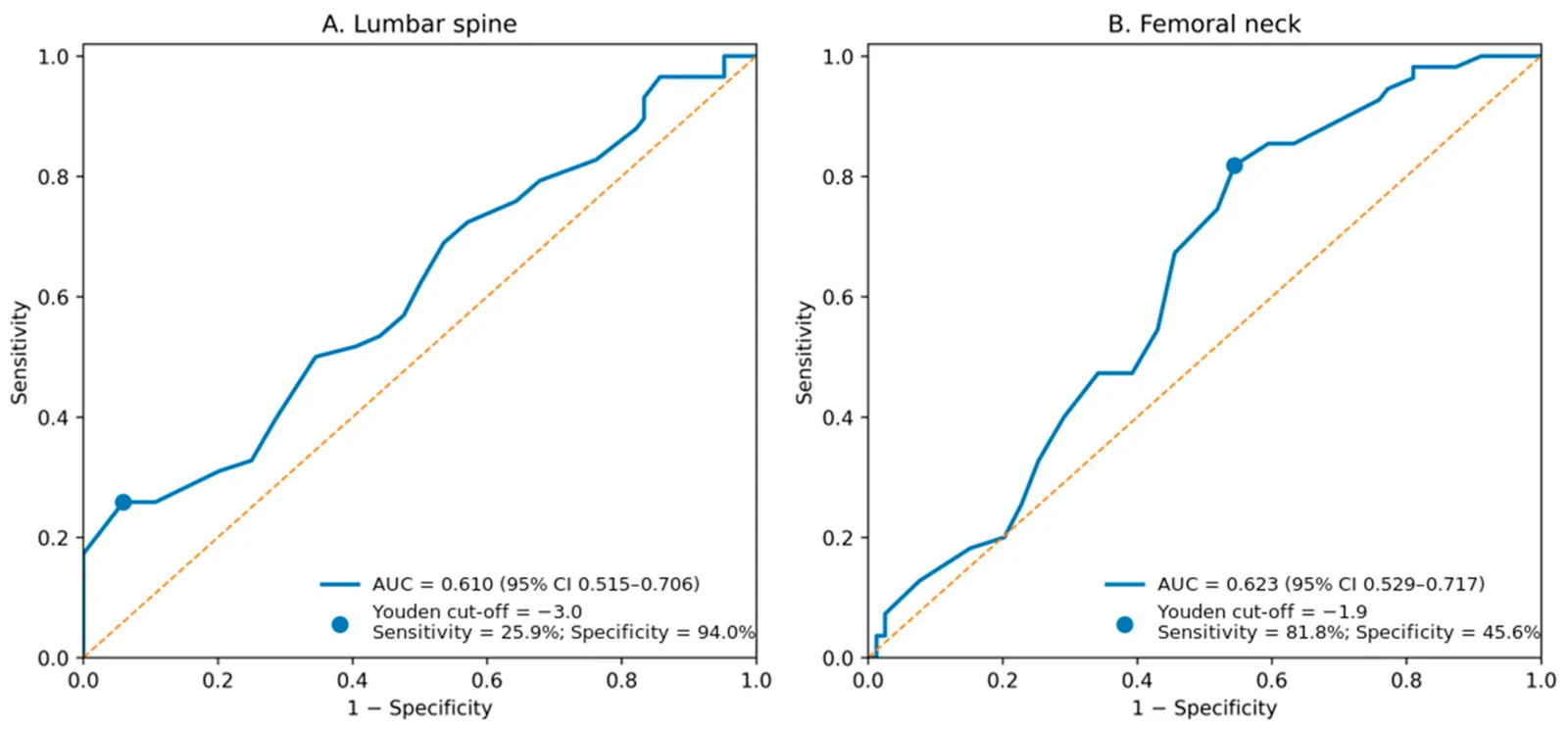
ROC curves of REMS T-score for the identification of DXA-defined osteoporosis at the lumbar spine (**A**) and femoral neck (**B**). The dashed diagonal line represents chance-level discrimination (AUC = 0.5).

ROC analysis for the diagnosis of osteoporosis showed an AUC of 0.610 (95% CI: 0.515–0.706) at the lumbar spine and 0.623 (95% CI: 0.529–0.717) at the femoral neck, indicating limited discriminatory performance. The optimal REMS T-score cut-off identified by the Youden index was −3.0 at the lumbar spine (sensitivity 25.9%, specificity 94.0%) and −1.9 at the femoral neck (sensitivity 81.8%, specificity 45.6%) (Figure 1).

The agreement between REMS and DXA diagnostic classifications evaluated with linearly weighted Cohen’s kappa coefficient was 0.225 at the lumbar spine and 0.183 at the femoral neck. Bland–Altman analysis showed a mean difference (REMS − DXA) in BMD of −0.023 g/cm^2^ at the lumbar spine, with 95% limits of agreement ranging from −0.301 to +0.255 g/cm^2^. At the femoral neck, the mean difference was +0.004 g/cm^2^, with 95% limits of agreement ranging from −0.216 to +0.224 g/cm^2^ (Supplementary Figure S2).

**Figure 2:**
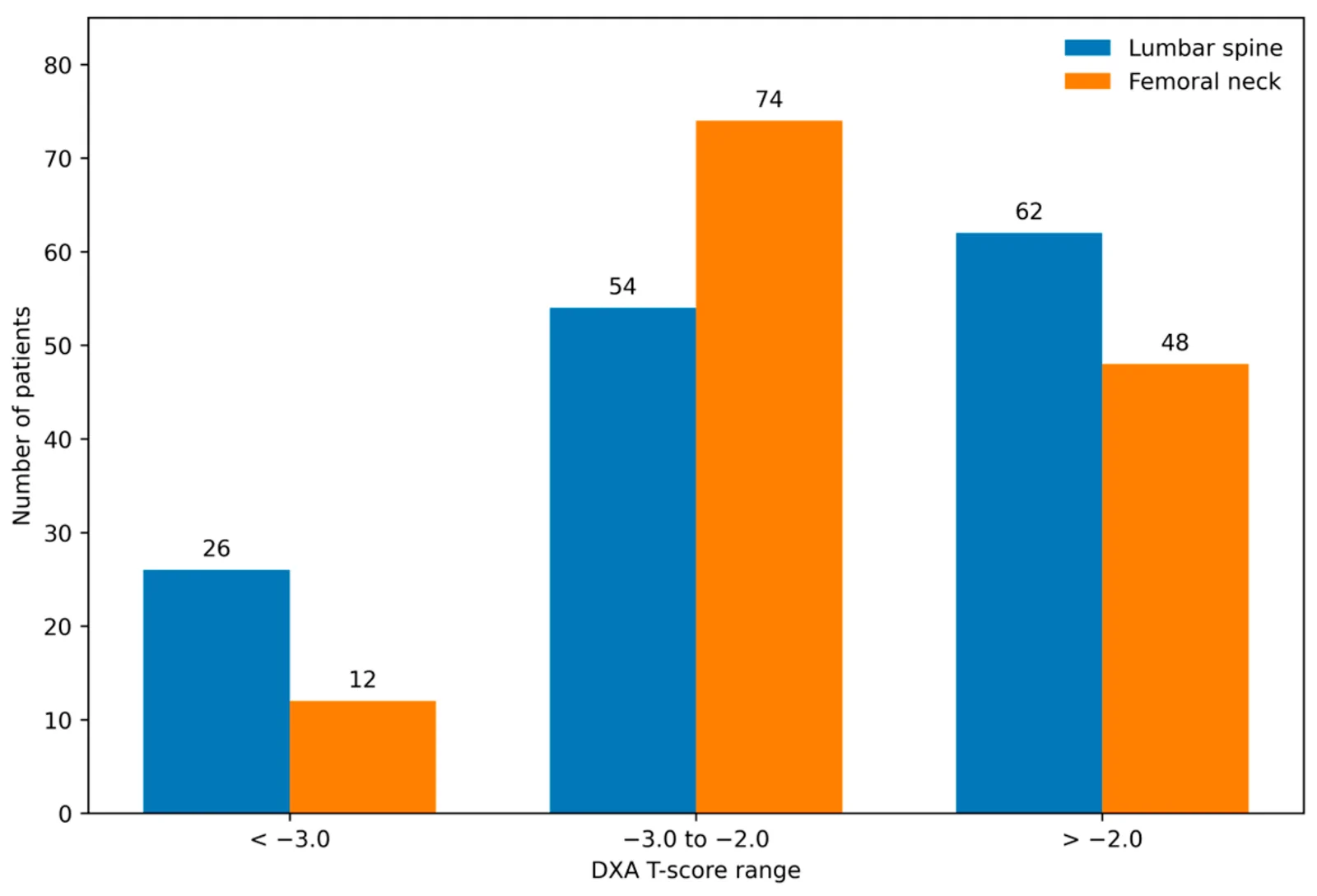
Distribution of patients stratified according to DXA T-score ranges at the lumbar spine and femoral neck.

Subjects were stratified according to DXA T-score ranges (Figure 2).

The distribution of discordant REMS–DXA classifications across DXA T-score ranges is presented in Table 4. At the lumbar spine, discordance rates were 50.0%, 42.6%, and 50.0% for DXA T-scores < −3.0, between −3.0 and −2.0, and > −2.0, respectively. Corresponding rates at the femoral neck were 66.7%, 54.1%, and 45.8%. No significant differences in discordance rate were detected across the T-score ranges at the lumbar spine (*p* = 0.692) or the femoral neck (*p* = 0.389).

Three-category cross-tabulations of REMS and DXA classifications were built to characterize the pattern of disagreement between the two techniques and are presented in Supplementary Table S2. At the lumbar spine, 67 of 142 paired examinations (47.2%) showed discordant classifications. The most frequent disagreements involved patients classified as osteopenic by REMS and osteoporotic by DXA (*n* = 28), followed by patients classified as osteoporotic by REMS and osteopenic by DXA (*n* = 22). At the femoral neck, 70 of 134 paired examinations (52.2%) showed discordant classifications, most frequently involving REMS osteopenia and DXA osteoporosis (*n* = 32), followed by REMS osteoporosis and DXA osteopenia (*n* = 22). Direct disagreement between normal bone density and osteoporosis was less frequent at both sites.

## 4. Discussion

Osteoporosis is a chronic skeletal disease characterized by reduced bone strength and an increased risk of fragility fractures [34]. In recent years, the therapeutic approach to osteoporosis has evolved substantially, moving from a predominantly antiresorptive strategy to a more personalized and sequential treatment [35,36]. Current evidence supports the early identification of patients at high fracture risk in order to optimize treatment strategies [37]. Although DXA remains the reference standard for the densitometric diagnosis of osteoporosis, in recent years REMS has generated growing interest as a potential adjunctive tool for the assessment of patients at risk. The present retrospective study evaluated the agreement and diagnostic performance of REMS compared with DXA in a real-world clinical cohort.

The main findings of the present study can be summarized as follows. Although no statistically significant differences in mean BMD or T-score values were detected between REMS and DXA at either anatomical site, agreement in individual diagnostic classification was limited. Linearly weighted Cohen’s kappa values were 0.225 at the lumbar spine and 0.183 at the femoral neck, while ROC analysis showed limited discriminatory performance for DXA-defined osteoporosis, with AUC values of 0.610 and 0.623, respectively.

The absence of statistically significant differences in mean BMD or T-score values does not establish equivalence or interchangeability between REMS and DXA. Consistently, Bland–Altman analysis showed a small mean bias at both anatomical sites but relatively wide 95% limits of agreement, indicating substantial variability in individual measurements. Since clinically acceptable limits of agreement were not predefined in the study protocol, the present data do not allow for a formal determination of whether the observed limits are clinically acceptable. Nevertheless, their width warrants caution when interpreting REMS and DXA measurements interchangeably in individual patients. At the individual level, measurement variability may be clinically relevant, especially when values lie close to diagnostic thresholds, because relatively small differences may result in different diagnostic classifications. However, the present data did not demonstrate a consistently higher discordance rate in the T-score range surrounding the osteoporosis threshold. Therefore, threshold proximity alone cannot explain the observed disagreement between REMS and DXA. These findings require careful clinical discussion and should be considered in the context of the existing literature on REMS. Several validation studies have reported substantially higher levels of agreement between REMS and DXA than those observed in the present analysis. In the multicenter European study by Cortet et al. [17], REMS demonstrated sensitivity and specificity values exceeding 90% for the identification of osteoporosis, with excellent correlations between DXA- and REMS-derived BMD measurements. Similar findings were reported by Di Paola et al. [20], who described REMS as a highly accurate technique for osteoporosis diagnosis. The lower diagnostic performance observed in this study may appear inconsistent with these reports. However, important methodological differences should be taken into account. Most published validation studies were conducted under highly controlled conditions and often included rigorous quality control procedures, dedicated operator training, and highly selected patient populations. In contrast, the present retrospective investigation reflects routine clinical practice and therefore represents a real-world assessment of REMS performance. Our cohort included eligible patients undergoing BMD evaluation for a range of endocrine conditions, reflecting the heterogeneity commonly encountered in daily endocrinological clinical settings. Such heterogeneity may contribute to increased measurement variability and reduced agreement compared with highly standardized validation environments.

Findings from real-world studies have been more heterogeneous. Medina et al. [26], in a real-world cohort of postmenopausal women, reported site-dependent agreement between REMS and DXA, with concordance correlation coefficients of 0.53 at the lumbar spine and 0.38 at the femoral neck. Amorim et al. [28], in a real-life study of 343 women, reported overall high diagnostic accuracy of REMS compared with DXA; however, only 227 lumbar spine and 238 hip examinations were considered acceptable for comparative analysis after exclusion of poor-quality acquisitions and other technical failures. These observations highlight that REMS performance in routine clinical settings may be influenced by anatomical site, patient selection and acquisition conditions.

Another important aspect deserving consideration concerns the interpretation of REMS false-positive results. When DXA is used as the reference standard, any patient classified as osteoporotic by REMS but not by DXA is, by definition, considered a false positive. However, such a classification reflects the methodological framework of the study rather than necessarily representing a true diagnostic error. It is well-established that fracture risk cannot be fully explained by BMD alone and that fragility fractures may occur in individuals who do not meet the densitometric criteria for osteoporosis. Furthermore, some factors, such as degenerative changes and calcifications, may determine an overestimation of BMD by DXA. REMS, based on the analysis of raw US radiofrequency signals, may capture structural information that differs from that provided by DXA [7,14,37,38]. Although it has been hypothesized that REMS may capture aspects of skeletal fragility not fully reflected by DXA-derived BMD, the present study was not designed to prove this hypothesis and does not provide evidence in its favor. Conversely, false-negative REMS classifications also warrant attention. Using DXA as the reference for densitometric osteoporosis, REMS failed to identify DXA-positive cases at the lumbar spine and at the femoral neck, with sensitivities of 50.0% and 40.0%, respectively. These findings indicate that, in this specific cohort, REMS used in isolation could miss a substantial proportion of patients meeting DXA criteria for osteoporosis.

The present findings suggest that REMS may be considered as a complementary rather than interchangeable or standalone approach to DXA. Although REMS cannot currently replace DXA, its portability and lack of ionizing radiation remain potential practical advantages and its clinical value should not be assessed solely in terms of agreement with DXA. Whether REMS provides additional information relevant to fracture-risk stratification in a real-world population requires prospective evaluation using fracture outcomes.

Several limitations should be acknowledged. First, the relatively small sample size and single-center retrospective design may limit the precision and generalizability of the findings, potentially introducing selection bias. Second, the study population included patients with different endocrine disorders, which may be associated with heterogeneous skeletal characteristics and may have influenced the observed diagnostic performance. The sample size did not allow for adequately powered subgroup analyses according to the underlying endocrine condition. Third, formal blinding between REMS and DXA operators was not implemented, and potential observer bias cannot be excluded. In addition, operator-specific intra- and inter-observer reproducibility was not assessed, limiting our ability to quantify measurement variability attributable to the operators. Furthermore, PPV and NPV may be influenced by the prevalence of osteoporosis in the study population and may therefore differ in populations with a different disease prevalence. Sensitivity and specificity are not directly determined by disease prevalence; however, their observed estimates may vary across populations because of spectrum and case-mix effects related to differences in disease severity, comorbidities, and other clinical characteristics. Consequently, our findings from patients attending a specialized endocrinology service may not fully reflect the general population. Finally, fracture outcomes and prospective follow-up were not available; therefore, the study cannot determine whether discordant REMS and DXA classifications differ in their ability to identify patients at increased fracture risk.

## 5. Conclusions

In this real-world study, REMS showed limited agreement with DXA for the assessment of osteoporosis. Although no statistically significant differences in mean BMD or T-score values were detected between the two techniques, diagnostic concordance at the individual level was limited. The diagnostic performance observed in this cohort does not support the use of REMS as a standalone screening method for DXA-defined osteoporosis. These findings suggest that REMS may represent a complementary tool for osteoporosis assessment but that the two techniques should not be considered interchangeable in this specific context. Further prospective studies including fracture outcomes are needed to clarify the clinical significance of REMS–DXA discordant cases and the potential role of REMS in identifying skeletal fragility not fully captured by DXA alone.

## Figures and Tables

**Table 1:** Demographic characteristics of the study population.

Cases, *n*	145
Age, years (mean ± SD)	63.8 ± 8.3
Female sex, *n* (%)	130 (89.7)
BMI, kg/m^2^ (mean ± SD)	24.5 ± 4.1
Primary	
Hyperparathyroidism, *n* (%)	7 (4.8)
Turner Syndrome, *n* (%)	5 (3.4)
Klinefelter Syndrome, *n* (%)	4 (2.8)
Cancer treatment-induced	
bone loss, *n* (%)	34 (23.4)

Continuous variables are expressed as mean ± standard deviation, categorical variables are expressed as frequencies and percentages.

**Table 2:** Bone mineral density, T-scores, and diagnostic classifications obtained by REMS and DXA at the lumbar spine and femoral neck.

	REMS	DXA	*p*-Value
**Lumbar spine**			
Evaluable examinations, *n*	143	144	
BMD (g/cm^2^), mean ± SD	0.816 ± 0.085	0.839 ± 0.146	0.059
T-score, mean ± SD	−2.15 ± 0.75	−1.95 ± 1.31	0.075
Osteoporosis, *n* (%)	59 (41.3)	59 (41.0)	
Osteopenia, *n* (%)	75 (52.4)	63 (43.8)	0.005
Normal bone density, *n* (%)	9 (6.3)	22 (15.3)	
**Femoral neck**			
Evaluable examinations, *n*	139	138	
BMD (g/cm^2^), mean ± SD	0.623 ± 0.087	0.618 ± 0.112	0.653
T-score, mean ± SD	−2.07 ± 0.77	−2.12 ± 0.93	0.541
Osteoporosis, *n* (%)	47 (33.8)	56 (40.6)	
Osteopenia, *n* (%)	76 (54.7)	68 (49.3)	0.408
Normal bone density, *n* (%)	16 (11.5)	14 (10.1)	

Data are expressed as mean ± standard deviation or number (percentage). Percentages for diagnostic categories were calculated using the number of evaluable examinations for each technique and anatomical site. Comparisons of mean BMD and T-score values were performed using paired observations.

**Table 3:** Diagnostic performance of REMS for identifying DXA-defined osteoporosis at the lumbar spine and femoral neck.

Parameter	Lumbar Spine	Femoral Neck
Paired examinations, *n*	142	134
True positives (TP), *n*	29	22
False positives (FP), *n*	29	23
True negatives (TN), *n*	55	56
False negatives (FN), *n*	29	33
Sensitivity, % (95% CI)	50.0 (37.5–62.5)	40.0 (28.1–53.2)
Specificity, % (95% CI)	65.5 (54.8–74.8)	70.9 (60.1–79.7)
PPV, % (95% CI)	50.0 (37.5–62.5)	48.9 (35.0–63.0)
NPV, % (95% CI)	65.5 (54.8–74.8)	62.9 (52.5–72.2)

Values are presented as numbers and percentages with 95% confidence intervals. DXA was used as the reference for densitometric osteoporosis classification. Analyses were restricted to paired evaluable examinations at each anatomical site. TP, true positive; FP, false positive; TN, true negative; FN, false negative; PPV, positive predictive value; NPV, negative predictive value.

**Table 4:** Distribution and rates of discordant REMS–DXA classifications according to DXA T-score ranges.

Lumbar spine			
DXA T-score range	Total paired examinations, *n*	Discordant classifications, *n*	Discordance rate, %
<−3.0	26	13	50.0
−3.0 to −2.0	54	23	42.6
>−2.0	62	31	50.0
Total	142	67	47.2
Femoral neck			
DXA T-score range	Total paired examinations, *n*	Discordant classifications, *n*	Discordance rate, %
<−3.0	12	8	66.7
−3.0 to −2.0	74	40	54.1
>−2.0	48	22	45.8
Total	134	70	52.2

Discordance was defined as any difference between REMS and DXA in the three-category classification of bone status (normal bone density, osteopenia, or osteoporosis). Discordance rates were calculated using the total number of paired evaluable examinations within each DXA T-score range as the denominator.

## Data Availability

The data are available from the corresponding author upon a reasonable request.

## Supplementary Materials

The following supporting information can be downloaded at https://www.mdpi.com/article/10.3390/diagnostics16213453/s1, Table S1: contingency tables for REMS classification of DXA-defined osteoporosis; Table S2: three-category cross-tabulations of REMS and DXA diagnostic classifications at the lumbar spine and femoral neck; Figure S1: patients flow diagram; Figure S2: Bland–Altman plots of BMD measurements obtained by REMS and DXA at the lumbar spine (A) and femoral neck (B).

## Abbreviations

The following abbreviations are used in this manuscript:

REMS	Radiofrequency Echographic Multi-Spectrometry
BMD	Bone Mineral Density
DXA	Dual-energy X ray Absorptiometry
PPV	Positive predictive value
NPV	Negative predictive value
ROC	Receiver operating characteristic analysis
SD	Standard deviation
US	Ultrasound
AUC	Area under the curve
