# Supplementary material for: Diagnostic Performance of Radiofrequency Echographic Multi Spectrometry Compared with Dual-Energy X-Ray Absorptiometry: A Real-World Study
Source: Diagnostics (Basel). 2026 Oct 22;16(21):3453. doi: 10.3390/diagnostics16213453 (PMC13762182; doi:10.3390/diagnostics16213453)

Table S1. Contingency tables for REMS classification of DXA-defined osteoporosis.

Lumbar spine — *n* = 142

REMS	DXA osteoporosis (+)	DXA osteoporosis (−)	Total
Osteoporosis (+)	29	29	58
Osteoporosis (−)	29	55	84
Total	58	84	142

Femoral neck — *n* = 134

REMS	DXA osteoporosis (+)	DXA osteoporosis (−)	Total
Osteoporosis (+)	22	23	45
Osteoporosis (−)	33	56	89
Total	55	79	134

Table S2. Three-category cross-tabulations of REMS and DXA diagnostic classifications at the lumbar spine and femoral neck.

Lumbar spine

REMS classification	DXA normal	DXA osteopenia	DXA osteoporosis	Total
Normal	7	1	1	9
Osteopenia	8	39	28	75
Osteoporosis	7	22	29	58
Total	22	62	58	142

Femoral Neck

REMS classification	DXA normal	DXA osteopenia	DXA osteoporosis	Total
Normal	6	7	1	14
Osteopenia	7	36	32	75
Osteoporosis	1	22	22	45
Total	14	65	55	134

Figure S1. Patient flow diagram.

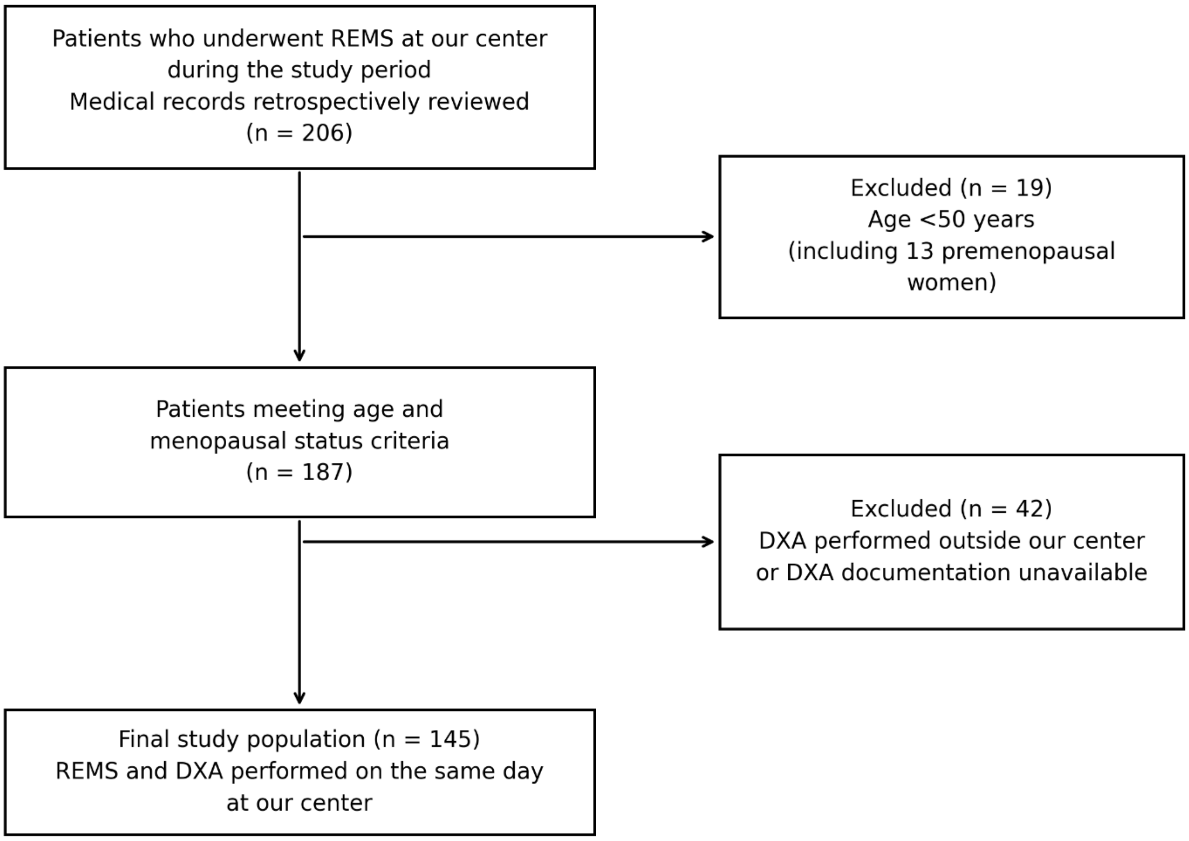

Figure S2. Bland–Altman plots of BMD measurements obtained by REMS and DXA at the lumbar spine (A) and femoral neck (B).

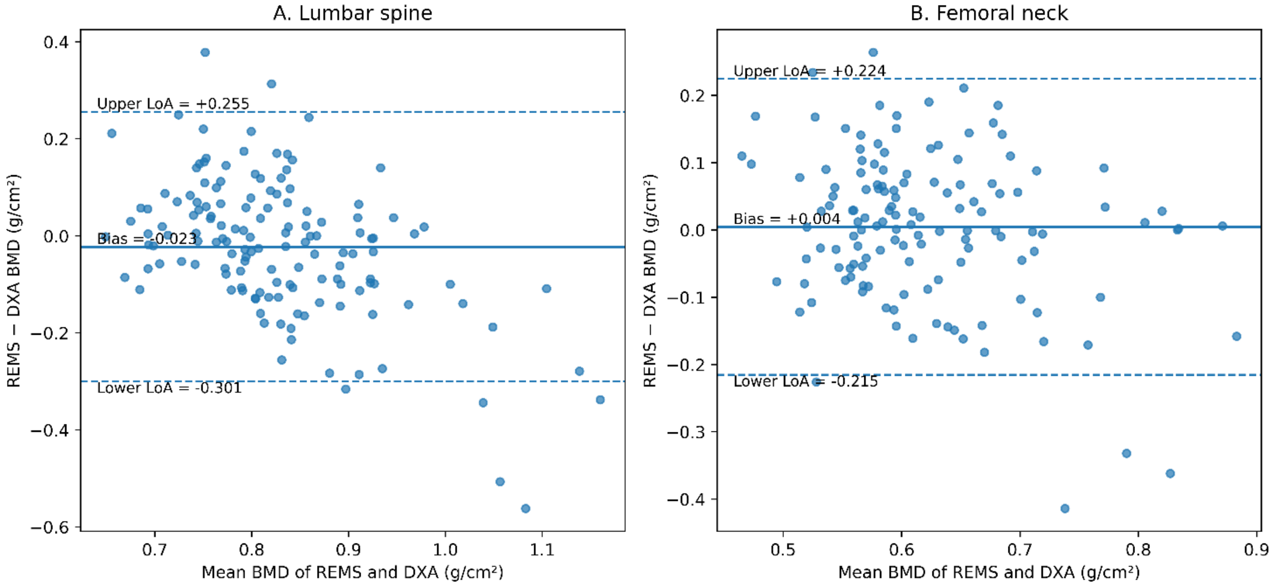

Supplement: Supplementary file 1 [file diagnostics-16-03453-s001.zip › diagnostics-4552355-supplementary.pdf]
